# Additions to *Neopestalotiopsis* (Amphisphaeriales, Sporocadaceae) fungi: two new species and one new host record from China

**DOI:** 10.3897/BDJ.10.e90709

**Published:** 2022-09-28

**Authors:** Yu-Ke He, Qi Yang, Ya-Ru Sun, Xiang-Yu Zeng, Ruvishika S. Jayawardena, Kevin D. Hyde, Yong Wang

**Affiliations:** 1 Center of Excellence in Fungal Research, Mae Fah Luang University, Chiang Rai 57100, Thailand Center of Excellence in Fungal Research, Mae Fah Luang University Chiang Rai 57100 Thailand; 2 Department of Plant Pathology, Agriculture College, Guizhou University, Guiyang, 550025, China Department of Plant Pathology, Agriculture College, Guizhou University Guiyang, 550025 China; 3 School of Science, Mae Fah Luang University, Chiang Rai 57100, Thailand School of Science, Mae Fah Luang University Chiang Rai 57100 Thailand

**Keywords:** morphology, Pestalotiod, phylogeny, taxonomy, Zingiberaceae

## Abstract

**Background:**

In this study, three *Neopestalotiopsis* taxa were identified, associated with leaves of *Zingiberofficinale*, *Elaeagnuspungens* and *Salaccazalacca*.

**New information:**

Based on morphology and multi–gene analyses of the internal transcribed spacer (ITS), beta-tubulin (TUB2) and translation elongation factor 1–alpha (TEF1), the five strains of *Neopestalotiopsis* represent two novel and one known species. They are introduced with descriptions, illustrations and notes herein.

## Introduction

Pestalotiod fungi distribute commonly as saprobes, pathogens and endophytes, which can cause a variety of plant diseases (Huanaluek et al. 2021). Most of this fungal group lack sexual morphs and only 13 species can reproduce the sexual stage (Maharachchikumbura et al. 2011, Nozawa et al. 2017). Pestalotioid fungi are placed in Sporocadaceae (Amphisphaeriales) (Jayawardena et al. 2019, Wijayawardene et al. 2020). Based on the conidia pigment colour, conidiophores and molecular phylogeny, *Neopestalotiopsis* was segregated from the old *Pestalotiopsis* genus (Maharachchikumbura et al. 2014a). *Neopestalotiopsis*, *Pestalotiopsis* and *Pseudopestalotiopsis* differ from each other by the colour of the conidial three median cells (Maharachchikumbura et al. 2014a). However, the delimitation of species, only through phenotypic characteristics, is difficult (Maharachchikumbura et al. 2016), thus morphological and phylogenetic approaches should be combined to determine the new taxa. Seventy-two species of *Neopestalotiopsis* are recorded in Index Fungorum (2022), but only forty-one species of *Neopestalotiopsis* are accepted, based on molecular data (Jayawardena et al. 2019). In this paper, two new species and a new Chinese record of *Neopestalotiopsis* found on *Zingiberofficinale* Rosc., *Elaeagnuspungens* Thunb. and *Salaccazalacca* (Gaertn.) Voss. in Zingiberaceae are described and illustrated.

## Materials and methods

### Sample collection and fungal strains isolation

Diseased fresh leaf samples were collected from *Z.officinale*, *E.pungens* and *S.zalacca* in Hainan Province, China in 2020. Fresh specimens were taken to the laboratory in paper envelopes. The strains were obtained using single spore isolation, following Senanayake (2020). Once the single spore germinated, it was transferred to potato dextrose agar (PDA) and cultured at room temperature (24°C).

### Morphological description

Microscopic slides were prepared with lactic acid and examined using an Axioscope 5 with Axiocam 208 colour (ZEISS, Oberkohen, Germany) at 1000× magnification. The morphology of fungi was photographed by the camera. Photo–plates were made by Adobe Photoshop CS6, with the Tarosoft (R) Image Frame Work programme being used for measurements. Herbaria materials were deposited in the Herbarium of the Department of Plant Pathology, Agricultural College, Guizhou University (HGUP). Cultures were deposited to the Culture Collection of the Department of Plant Pathology, Agriculture College, Guizhou University (GUCC) (Table 1). The taxonomic information of new species was submitted to MycoBank (http://www.mycobank.org).

### DNA extraction, PCR reaction and sequencing

The fresh mycelia were scraped off with a sterilised scalpel when colonies reached 80 mm in diameter. Genomic DNA was extracted using the Fungus Genomic DNA Extraction Kit (Biomiga GD2416), following the manufacturer’s instructions. Polymerase chain reactions (PCR) were performed in a 20 μl reaction volume: 1 μl of DNA template, 1 μl of each forward and reverse primers, 10 μl of 2× Bench Top^TM^Taq Master Mix and 7 μl double–distilled water (ddH_2_O). The partial internal transcribed spacer (ITS) rDNA was amplified with the primer pair ITS4/ITS5 (White et al. 1990), TEF1 was amplified with primers EF1–728f/EF2 (O’Donnell et al. 1998, Carbone and Kohn 1999) and the TUB2 was amplified with primers T1/Bt2b (Glass and Donaldson 1995, O'Donnell and Cigelnik 1997). PCR products were sequenced by using appropriate primers for amplification reactions by SinoGenoMax, Beijing. The obtained DNA sequences were submitted to GenBank to obtain their accession numbers (Table 1). DNA base differences on three loci between our strains and ex-type or representative strains of relative *Neopestalotiopsis* taxa are shown in Table 2.

### Phylogenetic analyses

The phylogeny was constructed by analyses from sequences of ITS, TEF1 and TUB2 sequence data. The fungal sequences were aligned by using the online version of MAFFT v. 7.307 (Katoh and Standley 2016) and edited by using the BioEdit programme (Hall 1999), using the SequenceMatrix 1.7.8 (Vaidya et al. 2011) to multi-source data merging. Ambiguous regions were excluded from analyses using AliView (Larsson 2014), gaps were treated as missing data and optimised manually with *Pestalotiopsisdiversiseta* Maharachch. & K.D. Hyde (MFLUCC 12–0287) as the outgroup (Table 2). Combined analyses of ITS, TUB2 and TEF1 sequence data were performed. Phylogenetic analyses were constructed by Maximum Likelihood (ML), Maximum Parsimony (MP) and Bayesian Posterior Probability (BYPP) methods; they were carried out as detailed in Dissanayake et al. (2020).

Maximum Parsimony analysis was performed with PAUP v. 4.0b10 (Swofford 2002), 1000 bootstrap replicates, using heuristic search on random stepwise addition and tree bisection reconnection (TBR). Maxtrees was set to 5000. For each tree generated, consistency index (CI), retention index (RI), tree length (TL), rescaled consistency index (RC) and homoplasy index (HI) were calculated.

The Maximum Likelihood analysis was performed using the CIPRES Science Gateway web server RAxML–HPC BlackBox (Stamatakis 2014) and 1000 rapid bootstrap replicates were run with the GTR+GAMMA model of nucleotide evolution.

Bayesian Posterior Probability analyses were performed by MrModeltest v.2.3 (Nylander et al. 2004) and MrBayes 3.2 (Ronquist et al. 2012) with the Markov Chain Monte Carlo (MCMC) method. The GTR model was selected as the best model for the TUB2. The MCMC runs were launched with four chains starting from random tree topology between 1,000,000–5,000,000 generations and sampling every 100 generations. The first 5000 samples were excluded as burn–in.

### Genealogical Concordance Phylogenetic Species Recognition (GCPSR) analysis

The phi-test incorporated in the SplitsTree software (Huson 1998, Huson and Bryant 2006) was used to test signals of recombination as described by Quaedvlieg et al. (2014). The evolutionary independence was revealed, using the GCPSR concept for the *Neopestalotiopsis* dataset with relevant taxa. In the pairwise homoplasy index (PHI), if a value below 0.05 was obtained, it provided evidence for the presence of significant recombination within a dataset. The test is proven to be a robust calculation and no previous knowledge about population history, recombination rate, mutation rate and rate heterogeneity across sites is necessary (Bruen et al. 2006).

## Taxon treatments

### Neopestalotiopsis elaeagni

Y.K. He & Yong Wang bis sp. nov.

8C7B700A-5EB9-578E-BE4D-BA002FCABC08

844750

#### Materials

- **Type status:** Holotype. **Occurrence:** recordedBy: Yu-ke He; occurrenceID: GUCC 21002; **Taxon:** scientificName: *Neopestalotiopsiselaeagni*; order: Amphisphaeriales; family: Sporocadaceae; genus: Neopestalotiopsis; **Location:** country: China; stateProvince: Hainan; locality: Haikou City, Leiqiong Haikou Volcano Cluster World Geopark; verbatimCoordinates: 109°39’ E, 20°13’ N; **Identification:** identifiedBy: Yu-ke He; dateIdentified: 2020; **Record Level:** collectionID: HGUP 10002

#### Description

Associated with the leaf blight of *Elaeagnuspungens* Thunb. Disease symptom: A large irregular scab on the leaves of *E.pungens*, light brown, edges dark brown to reddish-brown. The boundary of the scab was not obvious. There were many black, small and punctuate conidia on the scab. Sexual morph: not observed. Asexual morph (Fig. 2): Conidiomata dark, punctiform, scattered on the host scab, 110‒300 μm (n = 40), releasing black conidia. Conidiophores discrete to lageniform, hyaline, smooth– and thin–walled, 8‒13 × 2‒3 μm. Conidia 19‒25 × 4.5‒7 μm, fusiform to clavate, straight to slightly curved, 4–septate; basal cell obconic with a truncate base, hyaline or pale brown, smooth– and thin–walled, 3.5‒5 μm long; three median cells 12‒15 μm long, versicoloured, dark brown to light brown, septa and periclinal walls darker than the rest of the cell; second cell brown, 3.5‒5.5 μm long; third cell brown, 3‒5.5 μm long; fourth cell light brown 3.5‒5 μm long; apical cell 3‒5.5 μm long, hyaline, conic to acute, with 1–3 tubular appendages inserted at different loci, but in the same crest at the apex of the apical cell, unbranched, flexuous, 13‒30 μm long; most conidia have tubular appendages or single appendage in the basal cell, hyaline, unbranched, centric, 5‒7.5 μm long.

Culture characteristics: Colonies on PDA medium reaching 5‒5.5 cm diam. After 10 d at 24℃, the mycelium white, cottony, odourless, soft, without exudate and round with regular edges. Under the surface of hyphal layer, releasing conidia in a black, slimy mass. The reverse side of the culture dish is smooth and light yellow.

#### Etymology

*elaeagni*, in reference to the host genus (*Elaeagnus*) from which it was isolated.

#### Notes

Phylogenetically, the new species is sister to *Neopestalotiopsischrysea* (MFLUCC 12–0261), *Neopestalotiopsisumbrinospora* (MFLUCC 12–0285) and *Neopestalotiopsisasiatica* (MFLUCC 12–0286). However, *N.elaeagni* differed from *N.chrysea* by having shorter apical appendage (*N.elaeagni*: 13‒30 μm vs. *N.chrysea*: 22‒30 μm), differed from *N.umbrinospora* by having smaller conidia and shorter apical appendage (Conidia: *N.elaeagni*: 19‒25 × 4.5‒7 μm vs. *N.umbrinospora*: 19‒29 × 6‒8 μm; apical appendage length: *N.elaeagni*: 13‒30 μm vs. *N.umbrinospora*: 22‒35 μm) and differed from *N.asiatica* by having shorter apical appendage (*N.elaeagni*: 13‒30 μm vs. *N.asiatica*: 20‒30 μm) (Maharachchikumbura et al. 2012) (Table 3). According to the PHI analysis, our dataset showed a 1.0 value indicating no significant genetic recombination between our newly-introduced *Neopestalotiopsis* strains with other related taxa. Combined with morphology, phylogenetic analysis and PHI test results and we propose *N.elaeagni* as a novel species.

### Neopestalotiopsis zingiberis

Y.K. He & Yong Wang bis sp. nov.

3760484A-5AA5-549D-8F47-76D128D1C6C9

844751

#### Materials

- **Type status:** Holotype. **Occurrence:** recordedBy: Yu-ke He; occurrenceID: GUCC 21001; **Taxon:** scientificName: *Neopestalotiopsiszingiberis*; order: Amphisphaeriales; family: Sporocadaceae; genus: Neopestalotiopsis; **Location:** country: China; stateProvince: Hainan; locality: Haikou City, Wuzhishan Nature Reserve; verbatimCoordinates: 109°32’ E, 18°48’ N; **Identification:** identifiedBy: Yu-ke He; dateIdentified: 2020; **Record Level:** collectionID: HGUP 10001

#### Description

Associated with leaf blight of *Zingiberofficinale* Rosc. Disease symptom: A long oval to irregular, ring-like scab, light brown, edge reddish-brown, slightly sunken on adaxial surface. The boundary of the scab is obvious, with a narrow yellow halo around the scab. There are many black, small and punctuate conidia on the scab. Sexual state: unknown. Asexual morph (Fig. 3): Conidiomata is dark, oblate, scattered on the host scab, 104‒202 μm. Conidiophores discrete to lageniform, hyaline, smooth– and thin–walled, annellidicae, 12‒25 × 3‒6 μm (n = 40). Conidia 21‒31 × 6‒9.5 μm, fusiform to clavate, straight to slightly, 4–septate; basal cell obconic with a truncate base, hyaline or pale brown, smooth– and thin–walled, 3‒6 μm long; three median cells 15‒19 μm long, septa and periclinal walls darker than rest of the cell, versicoloured, wall rugose; second cell brown, 4‒6 μm long; third cell brown, 4‒7 μm long; fourth cell light brown 4‒6 μm long; apical cell 3‒5 μm long, hyaline, conic to acute, with 1‒3 tubular appendages insert at different loci, but in the same crest at the apex of the apical cell, unbranched, flexuous, 12‒15 μm long; most spores have no tubular appendages or single appendage, unbranched, centric, 0‒6 μm long.

Culture characteristics: Colonies on PDA medium reaching 8‒9 cm diam. after 15 d at 24℃, the mycelium is yellowish or white, soft and round with irregular edges. Under the surface of hyphal layer, releasing conidia in a black, slimy mass. Dark brown pigment is deposited on the bottom of the Petri dish.

#### Etymology

*zingiberis*, in reference to the host genus (*Zingiber*) from which it was isolated.

#### Notes

**Figure 1.**
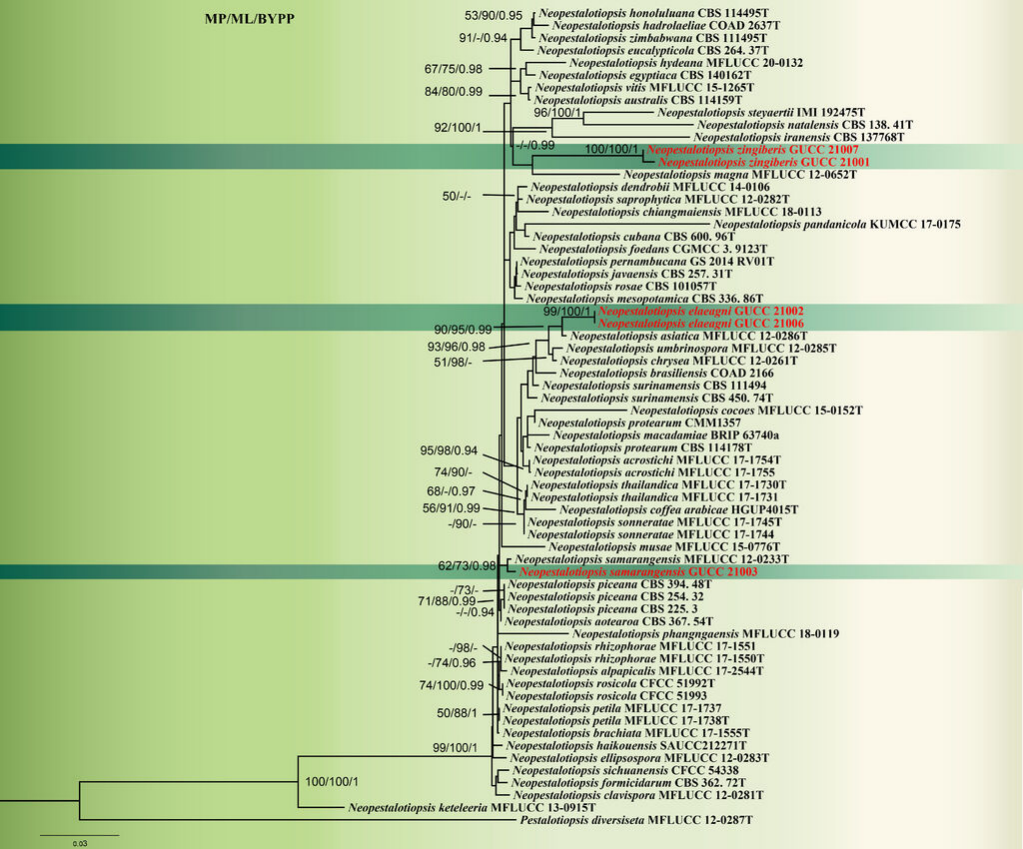
Consensus phylogram of 1,000 trees resulting from an RAxML analysis of the (ITS+TUB2+TEF1) alignment of the analysed *Neopestalotiopsis* sequences. *Pestalotiopsisdiversiseta* (MFLUCC 12–0287) is used as the outgroup taxon. The MP bootstrap values ≥ 50%, ML bootstraps ≥ 70% and Bayesian posterior probabilities ≥ 0.90 (MPBS/MLBS/PPBY) are given at the nodes. New collections obtained in this study are in red.

**Figure 2.**
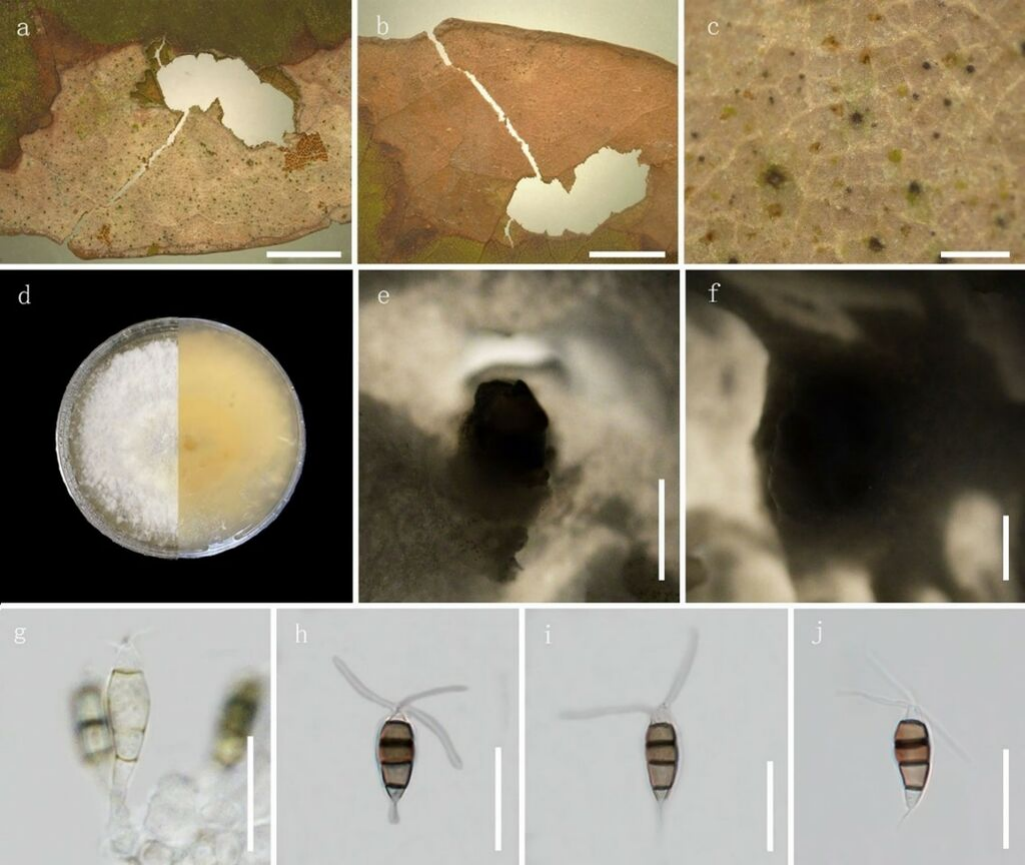
*Neopestalotiopsiselaeagni* (Specimen code: HGUP 10002). **a–c** Appearance on host surface; **d** Colony top view and reverse view; **e–f** Conidiomata on PDA; **g** Conidiogenous cells; **h–j** Conidia. Scale bars: **a–b** = 10 mm, **c** = 1 mm, **e–f** = 500 μm, **g–j** = 20 μm.

**Figure 3.**
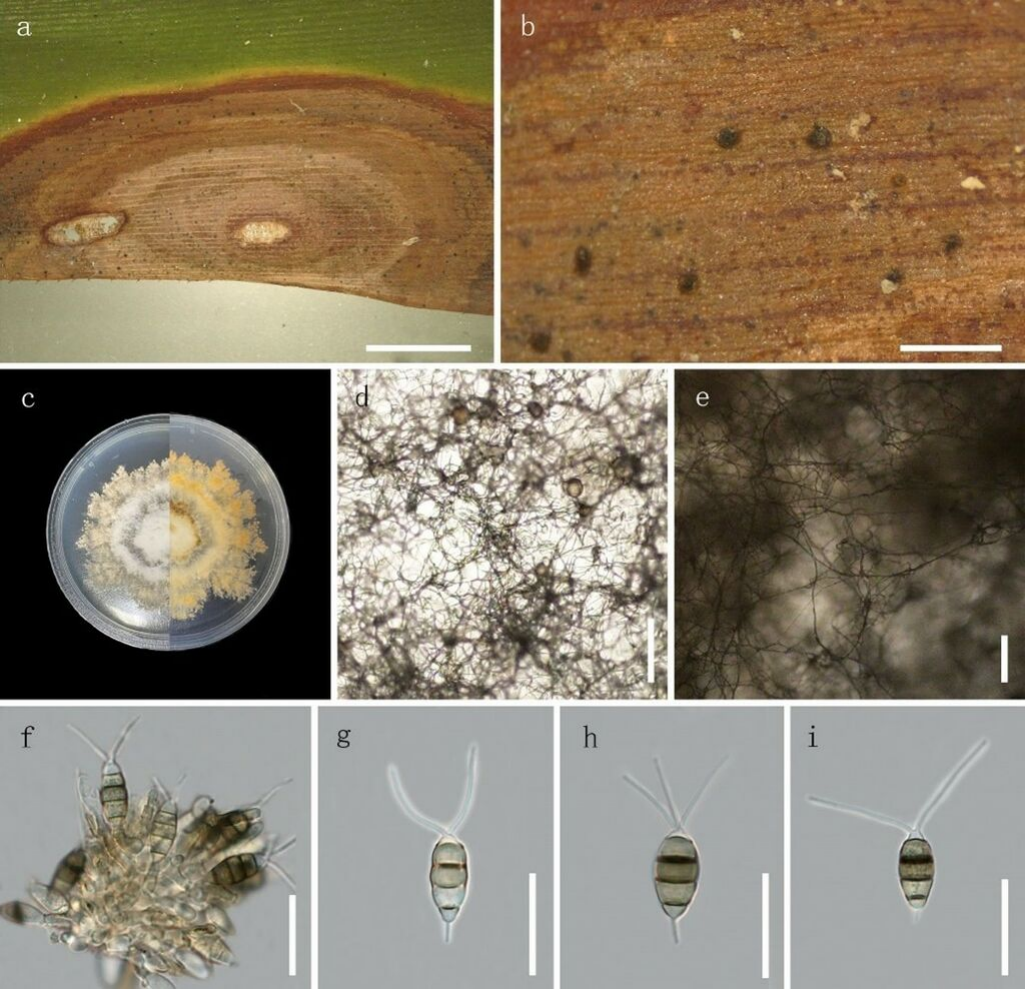
*Neopestalotiopsiszingiberis* (Specimen code: HGUP 10001). **a–b** Appearance on host surface; **c** Colony top view and reverse view; **d–e** Mycelium; **f** Conidiogenous cells; **g–i** Conidia. Scale bars: **a** =10 mm, **b** = 1 mm, **d–e** = 200 μm, **f–i** = 20 μm.

*Neopestalotiopsiszingiberis* (GUCC 21001) formed a distinct clade and sistered to *Neopestalotiopsismagna* (MFLUCC 12–0652) (Fig. 1). Morphologically, conidia of *N.zingiberis* (21‒31 × 6‒9.5 μm) are smaller than *N.magna* (42‒46 × 9.5–12 μm) and also differed by having branched, flexuous apical tubular appendages (Maharachchikumbura et al. 2014a) (Table 3). Thus, we propose *N.zingiberis* as a novel taxon.

### Neopestalotiopsis samarangensis

(Maharachch. & K.D. Hyde)

698327E5-F245-5B8A-A9B3-976100706183

809778

Neopestalotiopsis samarangensis (Maharachch. & K.D. Hyde) Maharachch., K.D. Hyde & Crous in Maharachchikumbura, Hyde, Groenewald, Xu & Crous, Stud. Mycol. 79: 147 (2014)

#### Materials

- **Type status:** Other material. **Occurrence:** recordedBy: Yu-ke He; occurrenceID: GUCC 21003; **Taxon:** scientificName: *Neopestalotiopsissamarangensis*; order: Amphisphaeriales; family: Sporocadaceae; genus: Neopestalotiopsis; **Location:** country: China; stateProvince: Hainan; locality: Haikou City, Xinglong Tropical Botanical Garden; verbatimCoordinates: 110°11’ E, 18°44’ N; **Identification:** identifiedBy: Yu-ke He; dateIdentified: 2020; **Record Level:** collectionID: HGUP 10003

#### Description

**Figure 4.**
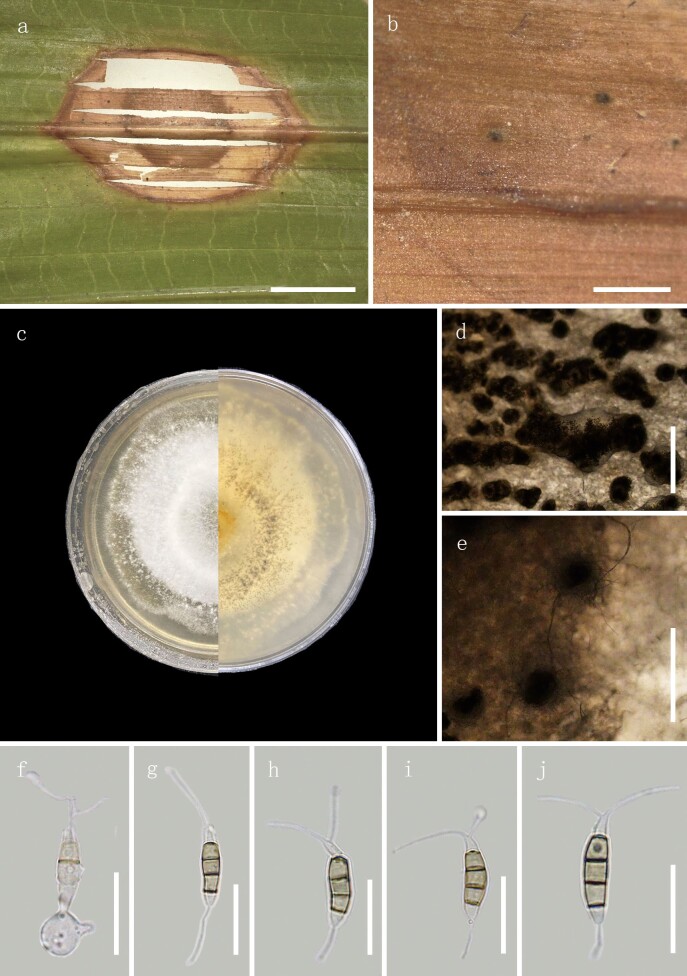
*Neopestalotiopsissamarangensis* (Specimen code: HGUP 10003). **a–b** Appearance on host surface; **c** Colony top view and reverse view; **d–e** Conidiomata on PDA; **f** Conidiogenous cells; **g–j** Conidia. Scale bars: **a** = 10 mm, **b** = 1 mm, **d–e** = 500 μm, **f–j** = 20 μm.

Associated with leaf spots of *Salaccazalacca* (Gaertn.) Voss. Disease symptom: a small oval scab, ring-like, the inner ring is light brown to dark brown and the outer ring is light brown, the boundary is obvious, dark brown. A few black, small, isolated and punctuate conidia irregularly distributed on the scab. Sexual state: unknown. Asexual morph (Fig. 4): Conidiomata is dark, oblate, scattered on the host scab, 70‒180 μm. Conidiophores discrete to lageniform or globular, hyaline, smooth– and thin–walled, simple and short. Conidia 18‒23 × 6‒7.5 μm, fusiform to clavate, straight to slightly, 4–septate; basal cell obconic with a truncate base, hyaline or pale brown, smooth– and thin–walled, 3.5‒5 μm long; three median cells 12.5‒15 μm long, light brown or hyaline, septa and periclinal walls darker than rest of the cell, wall rugose; second cell 4.5‒5.5 μm long; third cell 4‒5.5 μm long; fourth cell 5‒6 μm long; apical cell 3‒4.5 μm long, hyaline, conic to acute, with 1–2 tubular appendages inserted at different loci, but in the same crest at the apex of the apical cell, unbranched, flexuous, 12‒20 μm long. The spores have tubular appendages or single appendage, unbranched, centric, 3.5‒6 μm long.

Culture characteristics: Colonies on PDA medium reaching 4.5–5 cm diam. After 9 d at 24℃, odourless, without exudates, with black dots in the centre (conidiomata), the mycelium is white, soft and round with regular edges; reverse yellow to white. Under the surface of hyphal layer, releasing many conidia in a black, slimy mass.

#### Notes

Phylogenetically, isolated GUCC 21003 clustered with the ex-type strain of *N.samarangensis* (MFLUCC 12‒0233). In morphology, our strain is very similar to *N.samarangensis* (Maharachchikumbura et al. 2013). A comparison of DNA bases (Table 2) demonstrated that the differences between these two strains are minute. Therefore, we concluded that they are the same species, but occurring on different hosts (*N.samarangensis* GUCC 21003 on leaf of *Salaccazalacca* vs. *N.samarangensis* MFLUCC 12-0233 on *Syzygiumsamarangense*).

## Analysis

### Phylogenetic analysis

The final concatenated alignment comprised 1809 characters including 65 taxa. The combined dataset contained 1352 constant, 253 parsimony uninformative and 204 parsimony informative characters. According to different optimisation criteria, the tree topology was similar, so the individual datasets were congruent and could be combined. There were two equally parsimonious trees from MP analysis and we chose the best one to show the topology (Fig. 1) (TL = 855, CI = 0.680, RI = 0.651, RC = 0.442, HI = 0.320). *Neopestalotiopsiselaeagni* (GUCC 21002) is a sister taxon of *N.chrysea* and *N.umbrinospora* with high support (MP-BS = 90%/96% ML-BS = 96% BYPP = 0.98). *Neopestalotiopsiszingiberis* (GUCC 21001) is a sister taxon of *N.magna* (MFLUCC 12–0652) only with high BI support (PP = 0.96). GUCC 21003 was closer to the ex-type strain of *N.samarangensis* (MFLUCC 12-0233T) with high BI support (PP = 0.98). The base-pair differences amongst the three new collections are listed in Table 2. It showed that *N.elaeagni* (GUCC 21002), *N.chrysea* (MFLUCC 12-0261), *N.umbrinospora* (MFLUCC 12-0285) and *N.asiatica* (MFLUCC 12-0286), differ by only one character difference in the ITS region, 19-27 characters in TEF1 and 2-5 characters in TUB2. Between *N.zingiberis* (GUCC 21001) and *N.magna* (MFLUCC 12-0652), there were 16 character differences in the ITS region, three characters in TEF1 and 35 characters in TUB2. Between *N.samarangensis* (GUCC 21003) and *N.samarangensis* (MFLUCC 12-0233), there was only one character difference in the ITS, nine characters in TEF1 and two characters in TUB2.

## Discussion

In this study, we describe two new species and one new host record from China, namely *Neopestalotiopsiselaeagni*, *N.zingiberis* and *N.samarangensis*, based on morphological and phylogenetic analyses. For the morphology, we chose several indicators for the classification of *Neopestalotiopsis*, such as the size of conidia, the number and length of apical appendages and the basal appendage length (Maharachchikumbura et al. 2014a). For the phylogeny, we found that the different gene segments can distinguish the different inter-species relationships in *Neopestalotiopsis* (Table 2). However, some differences lacked significant variation to clearly distinguish the species of *Neopestalotiopsis*, such as the length and colour of the three median cells, the number of basal appendages and the ITS sequence data of *N.elaeagni* and *N.chrysea*. Therefore, we needed to combine the morphology and the phylogeny data to identify the new species.

China has reported 55 fungal diseases on 10 species of Zingiberaceae, including new diseases (Qi and Jiang 1994). However, most of the species have been identified, based on morphology alone. Most studies focused on the secondary products of fungi in Zingiberaceae and little research has been done on the diversity of fungi in Zingiberaceae (Taechowisan et al. 2003, Ginting et al. 2013, Anisha and Radhakrishnan 2017, Gupta et al. 2022). *Neopestalotiopsis* have been found on many different hosts and plant families (Maharachchikumbura et al. 2014b, Hyde et al. 2020), but few species have been found on Zingiberaceae in China. Therefore, in future work, comprehensive studies on Zingiberaceous *Neopestalotiopsis* will result in many more species being described in China.

We were unable to conduct the pathogenicity test in this research, although the *N.elaeagni* and *N.zingiberis* were isolated from the leaf spots. On the future work, similar to other relevant fields in mycology, it is necessary to identify the pathogenic taxa to the species level (Jayawardena et al. 2021), as it can help us to prevent diseases caused by them and reduce economic losses.

## Supplementary Material

XML Treatment for Neopestalotiopsis elaeagni

XML Treatment for Neopestalotiopsis zingiberis

XML Treatment for Neopestalotiopsis samarangensis

## Figures and Tables

**Table 1.** GenBank accession numbers of molecular phylogenetic analyses. Ex–type isolates are labelled with superscript T. The new isolates are in bold.

**Taxa**	**Strain number**	**Host**	**Country**	**ITS**	**TUB2**	**TEF1**	**Reference**
*Neopestalotiopsisacrostichi*	MFLUCC 17–1754^T^	*Acrostichumaureum*	Thailand	MK764272	MK764338	MK764316	Norphanphoun et al. 2019
*N.acrostichi*	MFLUCC 17–1755	*Acrostichumaureum*	Thailand	MK764273	MK764339	MK764317	Norphanphoun et al. 2019
*N.alpapicalis*	MFLUCC 17–2544^T^	*Rhyzophoramucronata*	Thailand	MK357772	MK463545	MK463547	Kumar et al. 2019
*N.aotearoa*	CBS 367.54^T^	*Canvas*	New Zealand	KM199369	KM199454	KM199526	Maharachchikumbura et al. 2014b
*N.asiatica*	MFLUCC 12–0286^T^	unidentified tree	China	JX398983	JX399018	JX399049	Maharachchikumbura et al. 2012
*N.australis*	CBS 114159T	*Telopea* sp.	Australia	KM199348	KM199432	KM199537	Maharachchikumbura et al. 2014b
*N.brachiata*	MFLUCC 17–1555^T^	*Rhizophoraapiculata*	Thailand	MK764274	MK764340	MK764318	Norphanphoun et al. 2019
*N.brasiliensis*	COAD 2166	*Psidiumguajava*	Brazil	MG686469	MG692400	MG692402	Bezerra et al. 2018
*N.chiangmaiensis*	MFLUCC 18–0113	*Pandanaceae*	Thailand	–	MH412725	MH388404	Tibpromma et al. 2018
*N.chrysea*	MFLUCC 12–0261^T^	dead leaves	China	JX398985	JX399020	JX399051	Maharachchikumbura et al. 2012
*N.clavispora*	MFLUCC 12–0281^T^	*Magnolia* sp.	China	JX398979	JX399014	JX399045	Maharachchikumbura et al. 2012
*N.cocoes*	MFLUCC 15–0152^T^	*Cocosnucifera*	Thailand	NR156312	–	KX789689	Norphanphoun et al. 2019
*N. coffea–arabicae*	HGUP4015^T^	*Coffeaarabica*	China	KF412647	KF412641	KF412644	Song et al. 2013
*N.cubana*	CBS 600.96^T^	leaf litter	Cuba	KM199347	KM199438	KM199521	Maharachchikumbura et al. 2014b
*N.dendrobii*	MFLUCC 14–0106	*Dendrobiumcariniferum*	Thailand	MK993571	MK975835	MK975829	Ma et al. 2019
*N.egyptiaca*	CBS 140162^T^	*Mangiferaindica*	Egypt	KP943747	KP943746	KP943748	Crous et al. 2015
*N.elaeagni*	HGUP10002 HGUP10004	*Elaeagnuspungens*, *Elaeagnuspungens*	China China	**MW930716** **ON597079**	**MZ683391** **ON595537**	**MZ203452** **ON595535**	**This study** **This study**
*N.ellipsospora*	MFLUCC 12–0283^T^	dead plant material	China	JX398980	JX399016	JX399047	Maharachchikumbura et al. 2012
*N.eucalypticola*	CBS 264.37^T^	*Eucalyptusglobulus*	–	KM199376	KM199431	KM199551	Maharachchikumbura et al. 2014b
*N.foedans*	CGMCC 3.9123^T^	unidentified mangrove plant	China	JX398987	JX399022	JX399053	Maharachchikumbura et al. 2012
*N.formicidarum*	CBS 362.72^T^	dead ant	Ghana	KM199358	KM199455	KM199517	Maharachchikumbura et al. 2014b
*N.hadrolaeliae*	COAD 2637^T^	*Hadrolaeliajongheana*	Brazil	MK454709	MK465120	MK465122	Freitas et al. 2019
*N.haikouensis*	SAUCC212271^T^	*Ilexchinensis*	China	OK087294	OK104870	OK104877	Zhang et al. 2022
*N.honoluluana* *N.hydeana*	CBS 114495^T^ MFLUCC 20–0132	*Telopea* sp. *Artocarpusheterophyllus*	USA Thailand	KM199364 MW266069	KM199457 MW251119	KM199548 MW251129	Maharachchikumbura et al. 2014b Huanaluek et al. 2021
*N.iranensis*	CBS 137768^T^	Fragaria×ananassa	Iran	KM074048	KM074057	KM074051	Song et al. 2013
*N.javaensis*	CBS 257.31^T^	*Cocosnucifera*	Indonesia	KM199357	KM199437	KM199543	Maharachchikumbura et al. 2014b
*N.keteleeria*	MFLUCC 13–0915^T^	*Keteleeriapubescens*	China	KJ503820	KJ503821	KJ503822	Song et al. 2013
*N.magna*	MFLUCC 12–0652^T^	*Pteridium* sp.	France	KF582795	KF582793	KF582791	Maharachchikumbura et al. 2012
*N.macadamiae*	BRIP 63740a	*Macadamia* sp.	Australia	KX186617	KX186656	KX186628	Akinsanmi et al. 2017
*N.mesopotamica*	CBS 336.86^T^	*Pinusbrutia*	Iraq	KM199362	KM199441	KM199555	Maharachchikumbura et al. 2014b
*N.musae*	MFLUCC 15–0776^T^	*Musa* sp.	Thailand	NR156311	KX789686	KX789685	Li et al. 2016
*N.natalensis*	CBS 138.41^T^	*Acaciamollissima*	South Africa	NR156288	KM199466	KM199552	Maharachchikumbura et al. 2014b
*N.pandanicola*	KUMCC 17–0175	*Pandanaceae*	China	–	MH412720	MH388389	Tibpromma et al. 2018
*N.pernambucana*	GS 2014 RV01^T^	*Vismiaguianensis*	Brazil	KJ792466	–	–	Maharachchikumbura et al. 2014b
*N.petila*	MFLUCC 17–1738^T^	*Rhizophoramucronata*	Thailand	MK764275	MK764341	MK764319	Norphanphoun et al. 2019
*N.petila*	MFLUCC 17–1737	*Rhizophoramucronata*	Thailand	MK764276	MK764342	MK764320	Norphanphoun et al. 2019
*N.phangngaensis*	MFLUCC 18–0119	*Pandanaceae*	Thailand	MH388354	MH412721	MH388390	Tibpromma et al. 2018
*N.piceana*	CBS 394.48^T^	*Picea* sp.	UK	KM199368	KM199453	KM199527	Maharachchikumbura et al. 2014b
*N.piceana*	CBS 254.32	*Cocosnucifera*	Indonesia	KM199372	KM199452	KM199529	Maharachchikumbura et al. 2014b
*N.piceana*	CBS 225.3	*Mangiferaindica*	–	KM199371	KM199451	KM199535	Maharachchikumbura et al. 2014b
*N.protearum*	CBS 114178^T^	*Leucospermumcuneiforme* cv. *Sunbird*	Zimbabwe	JN712498	KM199463	KM199542	Maharachchikumbura et al. 2014b
*N.protearum*	CMM1357	–	–	KY549597	KY549632	KY549594	Maharachchikumbura et al. 2014b
*N.rhizophorae*	MFLUCC 17–1550^T^	*Rhizophoramucronata*	Thailand	MK764277	MK764343	MK764321	Norphanphoun et al. 2019
*N.rhizophorae*	MFLUCC 17–1551	*Rhizophoramucronata*	Thailand	MK764278	MK764344	MK764322	Norphanphoun et al. 2019
*N.rosae*	CBS 101057^T^	*Rosa* sp.	New Zealand	KM199359	KM199429	KM199523	Maharachchikumbura et al. 2014b
*N.rosicola*	CFCC 51992^T^	*Rosachinensis*	China	KY885239	KY885245	KY885243	Jiang et al. 2018
*N.rosicola*	CFCC 51993	*Rosachinensis*	China	KY885240	KY885246	KY885244	Jiang et al. 2018
*N.samarangensis*	MFLUCC 12–0233^T^	*Syzygiumsamarangense*	Thailand	JQ968609	JQ968610	JQ968611	Maharachchikumbura et al. 2012
*N.samarangensis*	HGUP10003	*Salaccazalacca*	China	**MW930717**	**MZ683392**	**MZ540914**	**This study**
*N.saprophytica*	MFLUCC 12–0282^T^	*Magnolia* sp.	China	KM199345	KM199433	KM199538	Maharachchikumbura et al. 2012
*N.sichuanensis*	CFCC 54338 = SM15-1^T^	*Castaneamollissima*	China	MW166231	MW218524	MW199750	Jiang et al. 2021
*N.sonneratae*	MFLUCC 17–1745^T^	*Sonneronataalba*	Thailand	MK764279	MK764345	MK764323	Norphanphoun et al. 2019
*N.sonneratae*	MFLUCC 17–1744	*Sonneronataalba*	Thailand	MK764280	MK764346	MK764324	Norphanphoun et al. 2019
*N.steyaertii*	IMI 192475^T^	*Eucalyptusviminalis*	Australia	KF582796	KF582794	KF582792	Maharachchikumbura et al. 2012
*N.surinamensis*	CBS 450.74^T^	soil under *Elaeisguineensis*	Suriname	KM199351	KM199465	KM199518	Maharachchikumbura et al. 2014b
*N.surinamensis*	CBS 111494	*Proteaeximia*	Zimbabwe	–	KM199462	KM199530	Maharachchikumbura et al. 2014b
*N.thailandica*	MFLUCC 17–1730^T^	*Rhizophoramucronata*	Thailand	MK764281	MK764347	MK764325	Norphanphoun et al. 2019
*N.thailandica*	MFLUCC 17–1731	*Rhizophoramucronata*	Thailand	MK764282	MK764348	MK764326	Norphanphoun et al. 2019
*N.umbrinospora*	MFLUCC 12–0285^T^	unidentified plant	China	JX398984	JX399019	JX399050	Maharachchikumbura et al. 2012
*N.vitis*	MFLUCC 15–1265^T^	*Vitisvinifera*	China	KU140694	KU140685	KU140676	Jayawardena et al. 2016
*N.zimbabwana*	CBS 111495^T^	*Leucospermumcunciforme*	Zimbabwe	JX556231	KM199456	KM199545	Norphanphoun et al. 2019
*N.zingiberis*	HGUP10001 HGUP10005	*Zingiberofficinale*, *Zingiberofficinale*	China China	**MW930715** **ON597078**	**MZ683390** **ON595538**	**MZ683389** **ON595536**	**This study** **This study**
*Pestalotiopsisdiversiseta*	MFLUCC 12–0287^T^	dead plant material	China	NR120187	JX399040	JX399073	Maharachchikumbura et al. 2012

**Table 2.** The differences of DNA bases on different gene regions between our strains. Our strains are in bold.

**Species**	**Strain number**	**ITS (1–568)**	**TEF1 (569–1520)**	**TUB2 (1521–1972)**
***Neopestalotiopsiselaeagni*** ^*^	GUCC 21002	0	0	0
*Neopestalotiopsischrysea*	MFLUCC 12–0261	1	27	2
*Neopestalotiopsisumbrinospora*	MFLUCC 12–0285	1	25	4
*Neopestalotiopsisasiatica*	MFLUCC 12–0286	1	19	5
***Neopestalotiopsiszingiberis*** ^*^	GUCC 21001	0	0	0
*Neopestalotiopsismagna*	MFLUCC 12–0652	16	3	35
***Neopestalotiopsissamarangensis*** ^*^	GUCC 21003	0	0	0
*Neopestalotiopsissamarangensis*	MFLUCC 12–0233	1	9	2

**Table 3.** Comparison of conidia of *Neopestalotiopsis* species related to this study. Our strains are in bold.

**Species**	**Strain**	**Conidial size (μm)**	**Apical appendages**		**Basal appendage** **Length (μm)**
			Number	Length (μm)	
*N.chrysea*	MFLUCC 12–0261	20‒24 × 5.5‒7	3	22‒30	3–6
*N.umbrinospora*	MFLUCC 12–0285	19‒29 × 6‒8	1–3	22–35	5–7
*N.asiatica*	MFLUCC 12–0286	20‒26 × 5‒7	2–4	20–30	4–8
***N.elaeagni*** ^*^	GUCC 21002	19‒25 × 4.5‒7	1–3	13‒30	5‒7.5
	GUCC 21006				
*N.magna*	MFLUCC 12–0652	42‒46 × 9.5–12	2–4	16–26	11–15
***N.zingiber*** ^*^	GUCC 21001	21‒31 × 6‒9.5	1‒3	12‒15	0‒6
	GUCC 21007				
